# Correspondence

**Published:** 1901-06

**Authors:** 


### CORRESPONDENCE.

*Editor Atlanta Journal-Record of Medicine:*

DEAR DR. WOLFF.—Probably long before Christ the parable of the beam and the mote was popular, the fault is so ingrained in human nature. Exceptionally scarce is the instance of it in the *Medico-Legal Journal,* in the article by the ex-president of the Medico-Legal Society of New York, Albert Bach, Esq., "The Rehabilitation of the Medical Expert Witness." (March, 1901.)

To begin with, the heading of the article is extravagant. Has the medical expert so entirely lost his moral standing that a rehabilitation is necessary? We know well enough that the position of the medical expert is not as it ought to be, but is a reform to be worked altogether from the medical side? Mr. Bach would fain make us believe it. According to him the M.D. became untrue to his lofty calling, and with the sackcloth and ashes he has to go to court and say "*pater, peccavi!* henceforward I will be a good boy."

The reason Mr. Bach alleges is the mercenary character of the expert medical testimony. Owing to this the medical expert "is looked upon as an advocate of the side he has espoused for a money consideration" (l. c. p. 483), "and there is a great likelihood of this prejudice increasing in proportion to the amount of one's compensation for rendering the service." (*Ibid.*) Hence the grievous loss of moral tone in the medical profession. The doctor "does not hesitate to swear to anything to subserve the interest of his employer" (l. c. p. 486), and "judges and lawyers believe very little of what such witnesses swear to" (l. c. p. 483), and consequently these intrinsic representatives of the proudest impulses of the "development of the highest civilization" (l. c. p. 485) are moved in deep sympathy by the observation, that "Monetary greed thus becomes the controlling factor in the medical expert's career" (l. c. p. 483).

The love which the legal moiety of the medico-legal wreath bears to the medical chits is touching, perfectly olive-branched; moved by the inner serenity of the social yearnings peculiar to the forensic artists, they proffer from the deepest rock-bottom nucleus of their brotherly sentiment the counsel, "The medical profession should be eager to its utmost in the establishment of truth and right" (l. c. p. 480).

Now, what physician, and were his the proudest one among the first of names, but would bow in humility at such a behest, and we do not know where to direct more particularly our homage, to the perspicacity of the insight, or the perspicuity of the expression. But there is more behind to call for our admiration. Mr. Bach displays not only the generosity of the legal branch of the medico-legal fraternity by telling the medical juniors what they have to do to deserve the respect of the world, but gives counsel also *how* to do it, and here we must request the reader to prepare for a motion for unanimity in a vote of thanks to the magnanimous representative of the profession which is known all the world over as the model one when it comes to a disinterested standing-out for pure humanity. The infallible way which our jurisperitus proposes, the sure road road on which the medical profession can hope to regain the lost moral tone, strike anew the strayed from path of professional purity and prestige, and more especially be favored with a good record in the case-books of the gentlemen of the bar, is expressed in the terse sentence "Medicine should be the willing handmaid of the law" (l. c. p. 480).

There you are. Who will contradare to swear? Where is the fraternity which like that of the law may claim a past of unimpeachable, stainless, pristine purity? We must refrain, indeed, in trying to do its record full justice, to avail ourselves of our own linguistical resources. They fall markedly short of the requirements of the occasion. We must call in the pen of a poet, and we select our favorite exponent of human kind, Friedrich von Schiller, in the lines:

> "Dies Kind, kein Engel ist so rein,
> Lasst's Eurer Huld empfohlen sein."
> (This child, no angel is so pure,
> Let all your grace by it immure.)

Mr. Bach goes even further. The impartiality which is so peculiar to the lawyers, which comes to them naturally and is so infinitely promoted by their habits *in foro*, moves him to stretch out his hand to aid the medical profession in the struggle to regain the lost tone of morality and professional self-esteem. He goes so far as to admit, that there can be cases, a bare possibility, that "Certain members of the legal profession are also blamable for such conduct of the medical expert" (l. c. p. 484), and he directs his admonition likewise to the disciples of the hoodwinked girl with the scales and the sword, the sword, mind you, in the right, the scales in the left hand. Our jurisperitus says, "The keynote to such a relation as I advocate between the men of science and the men of law, is the honest desire to promulgate naught but scientific truth" (l. c. p. 484). But if we do not hesitate to abide ethically by the diplomatic concession, "they are all, all honorable," we must intellectually join issue with our highly esteemed mentor. If we applaud impulsively his warning, "The two professions should refuse to be party to any effort to dim the light of science" (l. c. p. 484), we strenuously protest against the way in which he proposes to bring such a desirable result about. He says, "Honesty may be induced in the medical expert who is inclined to be dishonest, by enacting laws providing punishment for dishonesty and unprofessional conduct before trial and at the trial of a cause" (l. c. p. 486). This proposition is not only a living monument of the utter ignorance of the jurisperiti of a certain class of what is scientific truth, but an affront to the medical profession, of all the learned professions the one which may justly boast of an extra exhibit of candid and perseverant effort of promoting the ends of humanity. Jurisprudence, the law, all through the weary centuries, as long as there is a history of civilisatory endeavor, has shown that he is practically a failure, theoretically a fallacy. The Roman historian, Tacitus, no less the great general and statesman, Julius Cæsar, expresses his surprise to have found in Germany without any laws order and noble sentiment, while in Rome either was with difficulty upheld by the most elaborate law-system and forensic ordination. And are we not at this very moment facing a crisis which makes the failure of the law flagrant to save us from it? It was the contamination of juridic Rome which frustrated the hope of a decaying world, to hail in the Teutonic on-march the germ of a better civilization than that of forensic sophistication. But yet in the legal profession the infatuation of incorrigible ignorance, to subject the culture of humanity, instead of to the spontaneity of virtuous impulse, to the deadening dullness of punishment and revenge.

Never, as long as we have had a history of civilization, the law has been genuinely scientific learning, neither the Roman law nor the German law, neither the common nor the statute law. But it was a terrible reality, no matter what it had to be called to designate it properly. It was there, an unmistakable exhibit of authority. If, however, there is nothing scientific in jurisprudence itself, scientific truth has a hold upon it. Up to the period of scholarly actuation of which usually the name of Francis Bacon is used as landmark, any pile of learning could pass off as science and scientific truth, which started from some more or less adventurous sentence, called fundamental, and originating in the vocabulating skill of imaginative, abstract temerity, proliferation of construction, or pusillanimity of submission, and early in this period it was the law which was prominent among this breed of wisdom, omnipotent, as it were. But a critical turn, the deep earnest of the adepts of the speculations of the medium age, made an inroad into this frivolity of slipshod scholarship; Roger Bacon, the namesake of Francis, but a couple of centuries his less illustrious, although in many respects greater predecessor, was among the first rebellious heads who refused to be guided by arbitrary doctrines and dogmatic lore, elbowed the authorities aside and made nature, man, the world, directly and independently his study. And this course was by the bolder ones of his successors followed up, the onmarch being so slow that it took a whole century before anatomical exploration was made the starting point of the study of man, and if there was a "learned profession," which was a clog to such progress, it was the legal profession, the honorables of bar and bench. The greatest among the scholars whose avocation was the law, were unanimous during this period, that there were old women with red eyes, to whose witchcraft was due the hailstorm which occurred in the fields, and the fire they kindled to burn them at the stake illumined the boon of mankind of civilization by punishment and revenge.

With the demonstration of the systemic circulation of the blood by William Harvey in the beginning of the 17th century, the long row of momentous discoveries began, of which our own age may claim the major part, and without misrepresentation, intentionally, or what we may term slander, nobody can deny, that the medical profession either had its share in the discoveries, or, no matter where the discoveries were made, took them up and availed itself of their spirit to render the assistance more effective, which it is the vocation of the medical practitioner to tender. But, lo! instead of earning applause from the legal ones of the medico-legal association, the medical profession is told, that if they want at all to require a stage of professional respectability; if a rehabilitation shall be made possible, the only way is, that "medicine should be the willing handmaid of the law."

It is always pitiable to see eyes, anatomically in good condition, psychologically deficient. If jurisprudence has the ambition to march at the head of intellectuality; if it wants not merely to abide by the kind of learning which it was before Roger and Francis Bacon's time the puerile passion to call science; if it has the honest aspiration to refer juridical subject-matter to scientific principles, not merely empty abstraction-husk, in clowny vocabulation-game, it must, like natural science, buckle down to the inductive method, first by careful observation of nature, of reality, make sure of the facts to go by, and then draw the admissible inferences, not start with a set opinion, and swear to dogmas resting on an unwarranted, perfectly arbitrary fabric of more or less wild thought-construction. Mr. Bach says, that the Roman axiom, "Justitia non novit patrem, nec matrem, solum veritatem spectat justitia," and the other one, "Lex non favet votis delicatorum," has much force. But why? Simply because "justitia" can club down all opposition. Why does Mr. Bach not add, "Fiat justitia, pereat mundus"? This would characterize the situation still better. But that is a sort of polemizing which renders the counsel, given to the medical profession, very suspicious, to avail itself of the law as a guide, to find the honorable path of truth and science. What is it the legal profession theoretically is based on? A most ridiculous absurdity; it is the fiction that with the promulgation of a law the knowledge of the same is spread among all the public which it is the intention to bind by its tenor. As a fact, the lawyers are themselves with no more than a problematic knowledge of it. From one end to the other the law is an uncouth upshot of the rough-and-tumble of the formation of political systems, and a makeshift at best. But the accommodation of the legal fraternity of a knock-down argument in the shape of clubs and bayonets, eventually of Gatling guns, of powder and shot, allows the hypothesis to be made to stay, and a fellow, even, that cannot read, is styled to have been made perfectly intimate with regard to the laws of the land. Coming to the push, a law is rarely explicit enough to pass the critique of the most humble police court, and if the superior court pronounces contra, there is no warrant in the world that it is not for reason of inferior understanding. But "Roma locuta est," and down comes the trap door, and the victim of "scientific jurisprudence" is hanged by the neck till he is dead. *Richelieu*, the greatest sage of legal capacities in the history of French despotism, declared, "Donnez-moi six lignes du plus honnête homme, et j'aurai de quoi le faire pendre" (Give me six lines in writing of the most honest fellow, and I will find cause to get him hanged), and in the United States there is no case of homicide, and were it the most dastardly murder, but there are courts in which an acquittal is by no means outside the limits of possibility. Our vernacular speaks therefore of the "waxen nose of the law."

Thus, how striking the cogency of reason in the counsel of the worthy brother of the legal department, "Medicine should be the willing handmaid of the law!"

And as to the ethical foundation of the science of the profession, which is holding out its helping hand to assist the medical expert to recuperate the lost moral prestige which he cannot be without, to be fairly introduced in the sacred halls of legal prosecution, the hallowed ground where tread the high-priests of social sanctification, the pious gentlemen of the bar and bench, it is now more than two thousand years already that by the latter the tenet is patronized, pleaded for and propagated, "Qui suo jure utitur neminem lædit" (who uses his right wrongs nobody), a Jesuitic maxim, although hatched long before there were Jesuits nominally, which in candid, plain English means, get the legislature into your hands and you can steal, murder, commit perjury legally to your heart's content, scientifically, ethically even, and go afterwards on a triumphal trip through the country; in short, enjoy it to be one of the rogues who are not in the penitentiary because they are craftsmen of the law, the scientifically moved law, which medicine is advised, for its salvation sake, to become the willing handmaid of a shrewd counsel, for sure, for if you get the hang of the law, the law does not get the hang of you.

Mr. Bach tells us, "Justice abhors darkness and seeks light" (l. c. p. 480). But why then always continue as a badge that hoodwinked the girl with the knife, which is the deplorable legacy of a pitiable period of the past? Why the persistent dunciad of dogmatism that was doomed at the first dawn of the inductive method of scientific research? Jurisprudence sins not only scientifically against the rules of the commonest kind of common sense, but empirically too. The legal profession does not avail itself of the host of discoveries on the vast domain of natural science, to go over the long list of its musty doctrines critically, but has an extra arrangement to frustrate a possibility of reflection by which some stray rays of the modern light of improved investigation might pierce the dust-covered *portière* of its antique domestication. With an involuntary persiflage Mr. Bach enjoins the medical profession to avoid the dangerous cliffs of party-pleading, and the forensic *hocus-pocus,* which by the law, as undignified anachronism, is foisted in the curriculum of social progress, is founded altogether upon the abuse which the generous heart of the New York medico-legal step-brother is anxious to save the medical moiety of. The judge, it is true, is directed to follow the maxim, one of the few good ones we inherited from the legal scoundrels in Rome, "audiatur et altera pars" (now let us listen to the other fellow). But is not the judge, and the jury to boot, delivered up virtually *de facto,* if not intentionally to the diabolical stratagem of party-counsel, which envenoms in the consecration of the legal fight the very peace which is ostentatiously the aim of this fight? Can there be anything more lunatic in the world, than to establish in the judge an institution of equity, and then provide for an adulteration of this institution of equity by instituting a contest, a mongrel joust, which iniquity, in stupid imitation of the old ordeal of the middle ages, first has over it? We thank Mr. Bach heartily for his warm advocacy of the research of truth. We are of his opinion that it is the greatest boon which we can hope for mankind. But so much more we are spellbound at his failure to see the monstrous absurdity of the constitution of partiality of the bar. The judge is expected to find the truth, or to come as near it as human ability may promise. But the bench is left in the lofty purpose. The counsel, one would say, are its *aides-de-camp*, but must play the part of marplots. The maxim of the judge, to listen to either side, must be slighted by the counsel. The judge is required to be impartial; he could not but be it lest he be not true. But the lawyer is compelled on the penalty of indirect capital punishment to side with one party, just as if only, thanks to the lying of the counsel, the truth could be found which the judge is expected to speak.

There may be personal reasons to antagonize the reform, which sporadically by a few lawyers has been recommended, to abolish the barrier of bar and bench. But no jurisperitus can allege scientific arguments against the reform. The chances of realizing incomes higher than the presidential salary would decrease considerably indeed. But the chances of seeing the legal profession in an ethically leading position, where hitherto it never as yet has been found, would increase considerably. The legal counsel being as impartial as the judge, not forced by their position as now, to hunt down justice for their client's sake, the truth would be found, and not only the truth practically regarding the case in hand, but also the truth about the law itself theoretically, something which is pitiably absent in the entire legal profession, Mr. Bach not excepted. The latter, with his professional brethren, is laboring under a huge mistake which dates from the classical period of Roman jurisprudence, and which the legal profession have had much care to inculcate in the laity. It is the much cherished but not the less grotesque error, that ever justice is the result of the law, or that ever the requisite of justice is the law. There is no more huge a fallacy than this. *Justice precludes the law.* If the parties observe justice, that means, as long as they are willing to abide by the rule, to view their right from the standpoint of the adversary, there is no occasion for law. The law fight is only for the unjust, for parties which, the one or the other, mostly both, are anxious to cut out of the boodle a bigger slice than behooves them. Hence the contest.

The law will never be ethically influential or reformatory so long as it is considered itself the arrangement which is to establish justice. Justice can never be propagated by the law. The law can only deprave it. There is only one agency which can propagate justice. This is *the public spirit.* But the law, the courts of law, can do much to create this public spirit, and they can do this by stamping the legal procedure a *disgrace*, for the parties who go and seek the right of law, because they are unwilling to observe justice anent their reciprocating opponents. This is the standpoint of truth and honor, and only if Mr. Bach lends a hand to reach such lofty purpose, he can hope to help realizing the ideal by which we trust he is moved at heart.

Possibly, in raising our accusation against jurisprudence, we must exempt all the single members of the profession. They are a prey to the old Adamite inheritance that we get the best counsel at the hands of our adversaries. So Mr. Bach is very noteworthy in his reasoning anent the privileged position of the physician in his medical attendance on the witness stand. But can he deny, perhaps, that all he says applies in a hundredfold degree to the legal advocate *in foro?* We must even make the statement, that it is a preposterous lie, when it is said that we have publicity of the law-procedure. When the people were granted this, it was done with a *reservatio mentalis.* What they have been given is a sham, an imposition. It does not include the conspiracy *sub rosa* of client and attorney, and thereby all other publicity is made illusionary.

But Mr. Bach, the eyes open, sees nothing of all that. Nor does his reasoning indicate that he is aware of the circumstance that jurisprudence long heretofore pleaded guilty in her case. In ancient Rome justice was bound upon the having of an *actio legis*. This was the indispensable legal means of attaining justice. The forum could not be approached otherwise. This led to monstrous consequences, practically, and the pretor, determined by business interest, gave an *actio utilis*, which invested with the right that justice refused, and so justice corrected the crippling by law. Similarly our courts of common law have been supplemented by the courts of equity, and whatever law we devise, it will always be subject to correction by the will to do good, and this will to do good it is an ominous mistake of the gentlemen of bar and bench to believe necessarily its promotion subject to the forensic niceties of punishment and revenge.

Exceptionally, we admit, the passing of laws has been very beneficent. But this were cases when the laws passed abolished bad former laws.

A MEDICAL ONE OF THE MED. LEG. FRAT.

